# Significant financial stress associated with 13-fold higher odds of having a heart attack

**Published:** 2018

Significant financial stress is associated with a 13-fold higher odds of having a heart attack, according to research presented at the 18th Annual Congress of the South African Heart Association.

‘The role of psychosocial factors in causing disease is a neglected area of study in South Africa, perhaps because there are so many other pressing health challenges such as tuberculosis and HIV,’ said lead author Dr Denishan Govender, associate lecturer, University of the Witwatersrand, Johannesburg.

‘The INTERHEART study showed that psychosocial factors are independently associated with acute myocardial infarction (heart attack) in Africa but as far as we are aware there are no other published local data,’ said last author Professor Pravin Manga, professor of cardiology, University of the Witwatersrand.

This study included 106 patients with acute myocardial infarction who presented to a large public hospital in Johannesburg. A control group of 106 patients without cardiac disease was matched for age, gender and race. All participants completed a questionnaire about depression, anxiety, stress, work stress and financial stress in the previous month. The Likert scale was used to grade the experience of each condition.

Regarding financial stress, patients were graded with no financial stress if they were coping financially; mild financial stress if they were coping financially but needed added support; moderate financial stress if they had an income but were in financial distress; and significant financial stress if they had no income and at times struggled to meet basic needs. Levels of psychosocial conditions were compared between groups and used to calculate associations with having a heart attack.

Self-reported stress levels were common, with 96% of heart attack patients reporting any level of stress, and 40% reporting severe stress levels. There was a three-fold increased risk of myocardial infarction if a patient had experienced any level of depression (from mild to extremely severe) in the previous month compared to those with no depression.

Both work stress and financial stress were associated with a higher risk of acute myocardial infarction. The odds of myocardial infarction was 5.6 times higher in patients with moderate or severe work stress compared to those with minimal or no stress. Patients with significant financial stress had a 13-fold higher odds of having a myocardial infarction.

Dr Govender said: ‘Our study suggests that psychosocial aspects are important risk factors for acute myocardial infarction. Often patients are counselled about stress after a heart attack but there needs to be more emphasis prior to an event. Few doctors ask about stress, depression or anxiety during a general physical and this should become routine practice, like asking about smoking. Just as we provide advice on how to quit smoking, patients need information on how to fight stress.’

Professor Manga said: ‘There is growing recognition that many developing countries are experiencing an increasing prevalence of chronic diseases of lifestyle such as myocardial infarction, and South Africa is no exception. Our study shows that psychosocial aspects are an area of cardiovascular prevention that deserves more attention.’

Dr David Jankelow, chairman of the SA Heart 2017 congress, commented: ‘We know that the depressed cardiac patient is at greater risk. We as clinicians need to identify them much earlier, so that they can be referred for appropriate intervention. Cardiac rehabilitation together with counselling and reassurance will play an important role as well.’

Professor Fausto Pinto, ESC immediate past president and course director of the ESC programme in South Africa, said: ‘Psychosocial factors including stress at work, depression and anxiety contribute to the risk of developing cardiovascular disease and having a worse prognosis. European prevention guidelines say that psychosocial risk-factor assessment should be considered in people with, or at high risk of, cardiovascular disease to identify possible barriers to lifestyle change or adherence to medication.’
